# Electrosprayed Metal Oxide Semiconductor Films for Sensitive and Selective Detection of Hydrogen Sulfide

**DOI:** 10.3390/s91109122

**Published:** 2009-11-17

**Authors:** Camelia Matei Ghimbeu, Martine Lumbreras, Joop Schoonman, Maryam Siadat

**Affiliations:** 1 LASC Groupe Capteurs, ISEA, University Paul Verlaine-Metz, 7 rue Marconi, 57070 Metz, France; E-Mails: lumbre@univ-metz.fr (M.L.); siadat@univ-metz.fr (M.S.); 2 Delft Institut for Sustainable Energy, Delft University of Technology, Julianalaan 136, 2628 BL Delft, The Netherlands; E-Mail: joops@standford.edu

**Keywords:** semiconductor metal oxide, electrostatic spray deposition, gas sensors, pollutant gases

## Abstract

Semiconductor metal oxide films of copper-doped tin oxide (Cu-SnO_2_), tungsten oxide (WO_3_) and indium oxide (In_2_O_3_) were deposited on a platinum coated alumina substrate employing the electrostatic spray deposition technique (ESD). The morphology studied with scanning electron microscopy (SEM) and atomic force microscopy (AFM) shows porous homogeneous films comprising uniformly distributed aggregates of nano particles. The X-ray diffraction technique (XRD) proves the formation of crystalline phases with no impurities. Besides, the Raman cartographies provided information about the structural homogeneity. Some of the films are highly sensitive to low concentrations of H_2_S (10 ppm) at low operating temperatures (100 and 200 °C) and the best response in terms of R_air_/R_gas_ is given by Cu-SnO_2_ films (2500) followed by WO_3_ (1200) and In_2_O_3_ (75). Moreover, all the films exhibit no cross-sensitivity to other reducing (SO_2_) or oxidizing (NO_2_) gases.

## 1. Introduction

The high toxicity of hydrogen sulfide, which has significant negative impacts on health and the environment, has attracted attention to the necessity of monitoring and controlling this gas. With a maximum allowed limit in the atmosphere of 10 ppm H_2_S, developing reliable sensors with high sensitivity and also selectivity towards other gases is a real challenge. Metal oxide semiconductors (MOS) have been extensively investigated fFor this purpose due to their simplicity, small dimensions and attractive pricepoint. Several types of metal oxide semiconductors [1-3] have been used as sensing material for different type of gases [4-7].

Concerning H_2_S detection, the literature shows that copper oxide, present in the SnO_2_ structure, greatly improves the sensitivity towards H_2_S and the selectivity to some reducing gases [8,9]. Good sensitivity to H_2_S has also been reported by using tungsten oxide films [10]. In general, other materials can be used for the detection of H_2_S, and their performance as gas sensors depends mainly on their textural, morphological and structural properties [11,12] and also by the presence of dopants or additives [13-15]. Hence, different techniques [16-18] are used to fabricate films or powders of MOS with desired characteristics which further allows one to achieve good quality sensors.

In this paper the electrostatic spray deposition technique was selected for the preparation of Cu-SnO_2_, WO_3_ and In_2_O_3_ thin films, due to its advantages such as simplicity and cost-effective set-up, ambient atmosphere operation and easy control of surface morphology by tuning the deposition parameters (temperature, time, flow rate,…). The film deposition process is described along with their morphological and structural characterizations by scanning electron microscopy (SEM), atomic force microscopy (AFM), X-Ray diffraction (XRD) and Raman Spectroscopy. The sensing performance of the films in the detection of H_2_S are studied as function of the operating temperature in order to determine the maximum response to H_2_S. In addition, the cross-sensitivity of the films to other possible interfering toxic gases (NO_2_ and SO_2_) is evaluated.

## 2. Results and Discussion

### 2.1. Morphology and Structure Characterizations

**Figure 1.**
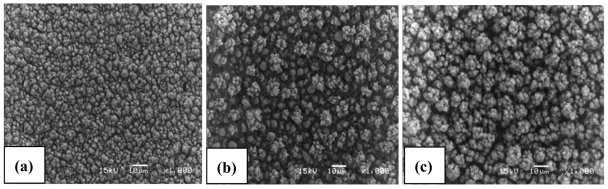
SEM pictures of (a) Cu-SnO_2_ (b) WO_3_ and (c) In_2_O_3_ films.

As evaluated by SEM pictures, the morphology of Cu-SnO_2_, WO_3_ and In_2_O_3_ films presented in Figure 1 shows a porous morphology comprising aggregates with uniform size distribution (2–5 μm for Cu-SnO_2_ and about 10 μm for In_2_O_3_). The WO_3_ films show variations in the size of the aggregates from 2 μm to 10 μm and this can be due to the lower deposition temperature (350 °C) compared to the deposition temperature of the other films (400 °C). The WO_3_ films deposited at 400 °C have a more developed porosity than the films prepared at 350 °C, but the adhesion to the substrate was poor, hence, a compromise had to be accepted. The morphology of the films deposited at a certain temperature depends mainly on the rate of evaporation, spreading, precipitation and decomposition reaction. For this reason different deposition parameters has been used for the preparation of the films (Table 1) in order to obtain porous morphology which plays an important role in the adsorption of the gas molecules [19]. The thickness of the films varies from 7 to 10 μm as determined by film cross-section.

Supplementary details about the deposition optimization process of the films are described elsewhere [20-22].

**Figure 2.**
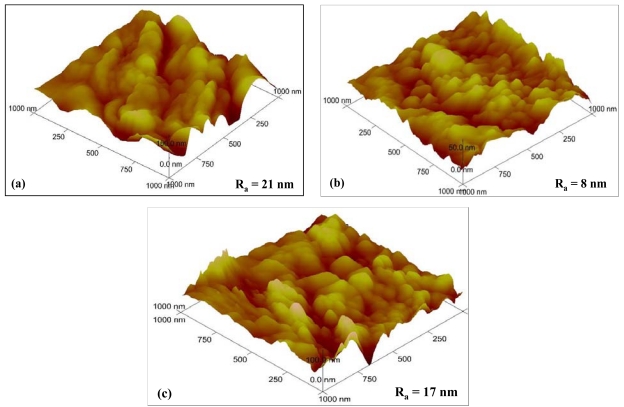
3D AFM topographies of (a) Cu-SnO_2_ (b) WO_3_ and (c) In_2_O_3_ films.

The topographic 3D views of Cu-SnO_2_, WO_3_ and In_2_O_3_ films are shown in Figure 2. The pictures, realised on a surface of 1 μm per 1 μm, provide information about the shape, the size of the grains and their distribution in the aggregates. Furthermore, by means of appropriate software the mean roughness (Ra) can be calculated. It can be seen that all the films have porous morphology comprising grains with sizes ranging from 100 to 250 nm. The roughness of the Cu-SnO_2_ seems to be the highest (21 nm), followed by In_2_O_3_ (17 nm) and WO_3_ (7 nm). Hence, no major differences between the films can be noted at this scale.

For a more precise evaluation of the roughness of the films, which could eventually provide a better comparison between the samples, it would be preferable to scan a larger surface. Unfortunately, this could not be successfully accomplished due to the specific morphology of the films, which present cauliflowers-like aggregates that are not connected between them at the surface, so, differences in the height of the aggregates and the “space” between them brought signal instabilities and consequently no good quality pictures could be obtained.

**Figure 3.**
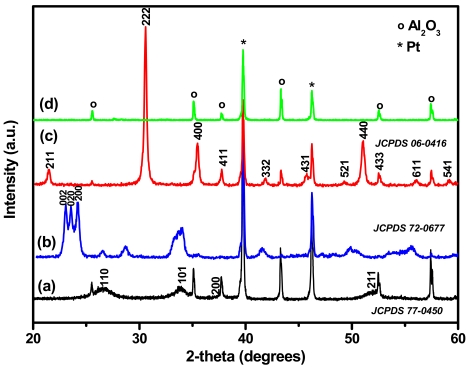
XRD patterns of (a) Cu-SnO_2_ (b) WO_3_ (c) In_2_O_3_ films and (d) Pt-Al_2_O_3_ substrate.

The crystallinity of the films was analysed by the XRD technique, as presented in Figure 3. The films exhibit crystalline phases with peaks corresponding to tetragonal rutile (Cu-SnO_2_), monoclinic (WO_3_) and cubic (In_2_O_3_) phases [20-22]. No supplementary peaks (except those of the substrate) are detected, proving the purity of the films. The average crystallite sizes are calculated according to Debye-Scherrer formula [23] and are about 7–10 nm for Cu-SnO_2_ and 20–30 nm for WO_3_ and In_2_O_3_. The small crystallite size of Cu-SnO_2_ is also suggested by their broad XRD peaks. For gas sensor applications the size of the crystallite is very important since improved sensitivity has been reported for materials which have crystallite size similar to twice of the space charge layer (2L) [12]. In our case the Cu-SnO_2_ has the closest value to 2L (6 nm).

**Figure 4.**
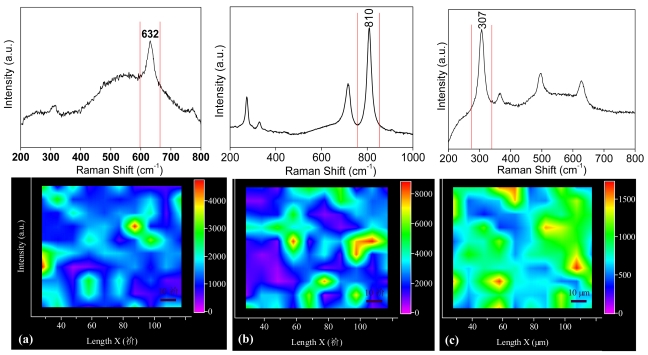
Raman spectra and cartographies of (a) Cu-SnO_2_ (b) WO_3_ and (c) In_2_O_3_ films.

Supplementary information concerning structural homogeneity of the films was provided by Raman spectroscopy studies. In Figure 4 the Raman spectra and cartographies of Cu-SnO_2_, WO_3_ and In_2_O_3_ are shown. All the present peaks are indexed to rutile SnO_2_ [24], monoclinic WO_3_ [25] and cubic In_2_O_3_ [26,27] in good accordance with the literature. Furthermore, these results validate the XRD ones with respect of the phase crystallization. To obtain the Raman cartographies approximately 100 spectra were collected for each film on a surface of 120 μm × 120 μm. The image was further obtained by the integration of the highest peak (632 cm^−1^ for Cu-SnO_2_, 810 cm^−1^ for WO_3_ and 307 cm^−1^ for In_2_O_3_) in each studied point.

It can be seen that the films are quite homogeneous in structure but they also present variations in the peak intensities which can be perhaps related to their morphology (especially to the grain size) and to the crystallinity. This type of Raman mapping gives an overall view of the surface structure of a material which is advantageous compared to the literature [28] where generally only one Raman spectrum is presented and sometimes the evaluation of a small structural variation is delicate to be exploited.

### 2.2. Gas Sensing Properties

**Figure 5.**
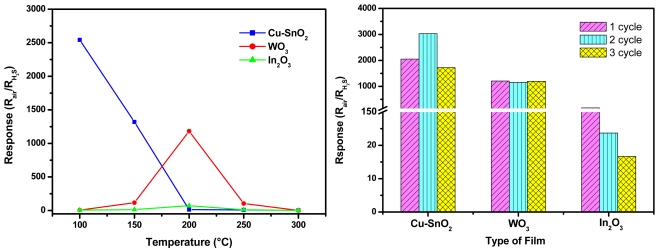
(a) Responses to 10 ppm H_2_S of Cu-SnO_2_, WO_3_ and In_2_O_3_ as a function of operating temperature and (b) Reproducibility of the film response to 10 ppm H_2_S at their optimum operating temperature.

Figure 5a presents the response (R_air_/R_H2s_) of the films to 10 ppm H_2_S as a function of the operating temperature. The maximum response (2500) is showed by Cu-SnO_2_ films at 100 °C. At this temperature the other films exhibit low response (about 6). They show better response (1200 for WO_3_ and 75 for In_2_O_3_) than Cu-SnO_2_ (13) at higher temperature (200 °C).

Comparing to the literature, the Cu-SnO_2_ prepared in this work presents higher response at lower operating temperature than some other Cu-SnO_2_ materials fabricated with different techniques [8]. In the same way, the performances of WO_3_ are superior to those reported in [29]. On the contrary, the response obtained in [10] is higher than that of our films, but with the disadvantage of a recovery process possible only by applying heating pulses at 250 °C. Concerning the In_2_O_3_ films in the detection of H_2_S, there are just few dedicated papers in the literature [30-32], and we can say that our film response is not remarkable.

The differences between the film responses studied in this work can be due to the nature of the studied oxide and their morphological and structural properties. The accepted mechanism for the detection of reducing gases consists in the reaction of chemisorbed oxygen species ( ${\text{O}}_{2}^{-}$, O^−^, O^2−^) on the film surface and the gas molecules (H_2_S). As a consequence of this reaction the electrons are released, the film resistance decreases (R_H_2_s_) and the response is improved. In addition, for Cu-SnO_2_ the influence of the copper dopant has to be taken into account since without dopant the sensitivity of SnO_2_ is very low [33]. In this direction several authors have proven by different techniques [34,35] the formation of CuS due to the reaction between Cu or CuO with H_2_S. The CuS metallic nature (low resistance) allows great sensor response to be achieved. The choice of a proper dopant for WO_3_ and In_2_O_3_ could beneficially improve their performance.

The reproducibility of the film response at their optimum operating temperature (Figure 5b) shows very good and satisfactory values for WO_3_ and Cu-SnO_2_, respectively. The In_2_O_3_ films present significant response differences between successive gas exposure cycles and this can be due to the incomplete recovery of the film resistance within the selected period of 60 minutes air purging. A good recovery of the resistance and implicitly of the response was be achieved by a long period (one night) of air exposure. Long recovery times for In_2_O_3_ have also been reported by other authors [36,37].

**Figure 6.**
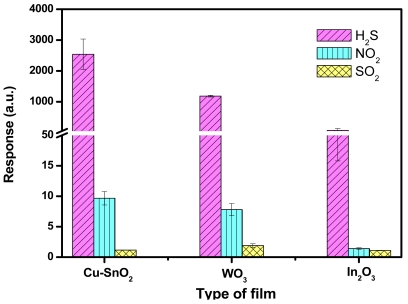
Responses of Cu-SnO_2_, WO_3_ and In_2_O_3_ to 10 ppm H_2_S, 1 ppm NO_2_ and 20 ppm SO_2_ at their optimum operating temperature.

The ability of detecting a desired gas from a mixture of gases is an important performance property of a sensor. For Cu-SnO_2_ the 100 °C optimum temperature was selected to study the cross-sensitivity to other reducing (SO_2_) and oxidising (NO_2_) gases, while for WO_3_ and In_2_O_3_ the 200 °C temperature was used. The selection of the temperature corresponds to the temperature offering the maximum response to H_2_S. As highlighted in Figure 6, all the films present significantly higher response for H_2_S than for NO_2_ or SO_2_. Hence, we can affirm that the films can provide selectivity in an eventually H_2_S detection from a mixture comprising the three studied gases (H_2_S, NO_2_ and SO_2_).

## 3. Experimental Section

### 3.1. Film Deposition Process

The films (Cu-SnO_2_, WO_3_ and In_2_O_3_) were deposited on Pt partially coated alumina using the ESD technique as presented in Reference [22]. The parameters used for the deposition of the films are described in Table 1. The precursors were dissolved in ethanol in order to obtain a 0.05 M solution which was atomisated by applying a high voltage (7-8 kV) between a metallic nozzle and a heated and grounded substrate. The formed aerosol (spray) comprises highly charged droplets which are directed to the substrate under an electrostatic force. At the impact, the droplets loose their charge, and then spreading, drying, and decomposition of the precursor solution occur. In this way a thin layer was formed on the substrate surface and to improve their crystallinity annealing treatment was performed. Several films of each composition have been prepared to validate the reproducibility of film deposition in terms of morphology, structure, homogeneity, and gas responses.

### 3.2. Characterization Techniques

The film morphology was studied using a JOEL JSM 580LV scanning electron microscope and the topography was evaluated with an atomic force microscope (Nanoscope IV-Multimode Veeco Instruments, USA) operating in a tapping mode regime. Raman measurements were performed at room temperature using a Jobin-Yvon microspectometer having a He-Ne excitation source (wavelength 632.8 nm). The sample surface was visualized by an optical microscope which allowed the selection of specific zone for structure evaluation. Raman spectra were recorded by scanning a part of the sample surface (120 μm × 120 μm) in 10 μm steps in both X and Y-axes. By integration of the desired Raman peak, the structural cartography of the surface was obtained.

### 3.3. Sensing Measurements

The gas sensing measurements were carried out in a closed quartz tube furnace. The temperature was measured using a type K thermocouple and was controlled by a PID temperature regulator (JUMO dTRON 16.1). The resistance measurements were performed using a two-point probe method with an electrometer (KEITHLEY 6514). The gas response of a film (S) is defined as the ratio of R_air_/R_gas_ for H_2_S and SO_2_ (reducing gases) and R_gas_/R_air_ for NO_2_ (oxidizing gas), where R_air_ represents the electrical resistance of the film in synthetic air and R_gas_ represents the resistance during the gas exposure.

To determine the temperature at which the H_2_S response was maximum, the films were exposed to 10 ppm H_2_S in a temperature range of 100 to 300 °C. Firstly the temperature was set to the desired value and the films were purged with synthetic air for 60 min. Next, the films were exposed to H_2_S for 30 min, followed by regeneration with synthetic air for 60 min. This cycle was repeated about four times during a day (in order to ensure the reproducibility). The responses of the films to 20 ppm SO_2_ and 1 ppm NO_2_ were evaluated at the selected operating temperature (temperature at which the film shows the maximum response).

The concentration of gases was fixed by adjusting the flow rates of a target gas and a carrier gas (synthetic air) in the way to maintain a constant total flow rate of 100 mL/min. The gas bottles were provided by Air Products (France) and the concentration of the gases was controlled using mass flow controllers (MFC, BROOKS Instruments, 5850 TR).

## 4. Conclusions

This paper describes the deposition of metal oxide semiconductor films of Cu-SnO_2_, WO_3_ and In_2_O_3_ using the electrostatic spray deposition technique and their use in the detection of hydrogen sulfide. It has been shown that this simple and cost-effective technique allows the fabrication of films with desired characteristics for gas sensor applications, *i.e.*, porous and homogeneous morphology, good crystallinity, crystallite size in the nanometer range and purity. These highlighted characteristics can afford a high sensitivity of our films to H_2_S with a maximum response achieved by Cu-SnO_2_ (2,500) and followed by WO_3_ (1,200) and In_2_O_3_ (75). In addition, their low operating temperatures (100 °C and 200 °C) makes them attractive from a practical and energy economy point of view. For measurements in real atmospheres, several gases can be present at the same time, so, the selectivity of a sensor is crucial in order to avoid interferences between different gases. With respect of this aspect no cross-sensitivity of all the studied films to SO_2_ or NO_2_ was found.

## Figures and Tables

**Table 1.** Experimental parameters used for the film deposition.

**Film Type**	**Precursor**	**Deposition Temperature (°C)**	**Deposition Time (h)**	**Flow Rate (mL/h)**	**Annealing Temperature (°C)**
Cu-SnO_2_ (1% Cu)	SnCl_4_·4H_2_O Cu(NO_3_)_2_·2.5H_2_O	400	1	2	550
WO_3_	W(C_2_H_5_O)_6_	350	1	1	500
In_2_O_3_	InCl_3_	400	1	1.5	500
